# Evidence of bisphosphonate-conjugated sitafloxacin eradication of established methicillin-resistant *S. aureus* infection with osseointegration in murine models of implant-associated osteomyelitis

**DOI:** 10.1038/s41413-023-00287-4

**Published:** 2023-10-18

**Authors:** Youliang Ren, Jason Weeks, Thomas Xue, Joshua Rainbolt, Karen L. de Mesy Bentley, Ye Shu, Yuting Liu, Elysia Masters, Philip Cherian, Charles E. McKenna, Jeffrey Neighbors, Frank H. Ebetino, Edward M. Schwarz, Shuting Sun, Chao Xie

**Affiliations:** 1 Center for Musculoskeletal Research, University of Rochester Medical Center, Rochester, NY 14642 USA; 2 Department of Orthopaedics, University of Rochester Medical Center, Rochester, NY 14642 USA; 3 Department of Pathology and Center for Advanced Research Technologies, University of Rochester Medical Center, Rochester, NY 14642 USA; 4 BioVinc, LLC, Pasadena, CA 91107 USA; 5 Department of Chemistry, University of Southern California, Los Angeles, CA 90089 USA; 6 Department of Pharmacology, Pennsylvania State University, Hershey, PA 17033 USA; 7 Department of Chemistry, University of Rochester, Rochester, NY 14642 USA

**Keywords:** Bone, Bone quality and biomechanics

## Abstract

Eradication of MRSA osteomyelitis requires elimination of distinct biofilms. To overcome this, we developed bisphosphonate-conjugated sitafloxacin (BCS, BV600072) and hydroxybisphosphonate-conjugate sitafloxacin (HBCS, BV63072), which achieve “target-and-release” drug delivery proximal to the bone infection and have prophylactic efficacy against MRSA static biofilm in vitro and in vivo. Here we evaluated their therapeutic efficacy in a murine 1-stage exchange femoral plate model with bioluminescent MRSA (USA300LAC::lux). Osteomyelitis was confirmed by CFU on the explants and longitudinal bioluminescent imaging (BLI) after debridement and implant exchange surgery on day 7, and mice were randomized into seven groups: 1) Baseline (harvested at day 7, no treatment); 2) HPBP (bisphosphonate control for BCS) + vancomycin; 3) HPHBP (hydroxybisphosphonate control for HBCS) + vancomycin; 4) vancomycin; 5) sitafloxacin; 6) BCS + vancomycin; and 7) HBCS + vancomycin. BLI confirmed infection persisted in all groups except for mice treated with BCS or HBCS + vancomycin. Radiology revealed catastrophic femur fractures in all groups except mice treated with BCS or HBCS + vancomycin, which also displayed decreases in peri-implant bone loss, osteoclast numbers, and biofilm. To confirm this, we assessed the efficacy of vancomycin, sitafloxacin, and HBCS monotherapy in a transtibial implant model. The results showed complete lack of vancomycin efficacy while all mice treated with HBCS had evidence of infection control, and some had evidence of osseous integrated septic implants, suggestive of biofilm eradication. Taken together these studies demonstrate that HBCS adjuvant with standard of care debridement and vancomycin therapy has the potential to eradicate MRSA osteomyelitis.

## Introduction

Chronic bone infections from *Staphylococcus aureus* (*S. aureus)* present a major challenge for orthopedics, as clinical studies have shown that standard of care (SOC) surgery with antibiotic therapy only results in a ~40% cure rate and outcomes from methicillin-resistant *S. aureus* (MRSA) are more severe.^1^ These clinical failures are largely due to recalcitrant biofilm bacteria, which are now known to exist in four distinct forms: 1) intracellular bacteria, 2) bacteria within glycocalyx on the implant and necrotic tissue, 3) Staphylococcus abscess communities (SACs) and 4) bacteria within the osteocyte-lacuno-canalicular network (OLCN) of cortical bone.^2–4^ While SOC debridement, irrigation, and conventional antibiotic therapy are effective against planktonic bacteria and some forms of biofilm, animal model studies demonstrated that this intervention has no efficacy against SACs and bacteria within the OLCN.^5,6^ It has also been demonstrated that the combination of high dose local and systemic antibiotic therapy fails to achieve the minimum biofilm eradication concentration (MBEC) within the OLCN compartment, likely due to unfavorable pharmacokinetics, resulting in persistent infection of susceptible bacterial strains.^7^

To overcome the pharmacokinetic limitations of SOC antibiotics, we pioneered bisphosphonate-conjugated antibiotics (BCA), which utilize a “target and release” mode of drug delivery to sites of bone infection.^8–10^ In this strategy non-nitrogen-containing bisphosphonates, which possess a high affinity to hydroxyapatite at resorbing bone surfaces while having negligible effects on bone cells, are conjugated to SOC antibiotics via a semi-labile chemical linkage that is stable in the bloodstream. With this approach, antibiotics with potency against biofilm bacteria can be specifically targeted to the bone-bacteria interface and kill the pathogens following acid and/or enzymatic cleavage of the linkage, releasing active antibiotic at the diseased bone surface.^9^ In support of this concept, we previously demonstrated that systemic delivery of a fluorescent bisphosphonate probe binds to the bone surface adjacent to bacteria in a murine model of *S. aureus* osteomyelitis, confirming bone targeting.^9^

To identify an ideal candidate antibiotic to conjugate with a bisphosphonate, we conducted a large screen of available FDA-approved drugs.^11^ These antibiotics were tested for their efficacy against *S. aureus* biofilm bacteria and small colony variants, which model the bacteria within the OLCN of infected bone. These experiments demonstrated that sitafloxacin was the most potent among these bactericidal drugs against both methicillin-sensitive *S. aureus* (MSSA) and methicillin-resistant *S. aureus* (MRSA) in vitro and in vivo.^11^ The results also showed that bisphosphonate-conjugated sitafloxacin (BCS) and hydroxybisphosphonate-conjugated sitafloxacin (HBCS) kill *S. aureus* in vitro and in vivo, as evidenced by permeabilized cell wall morphology imaged by scanning electron microscopy (SEM) and transmission electron microscopy (TEM).^9,10^ As SOC antibiotics are highly effective as prophylaxis for elective surgery (e.g., infection rates for primary total joint replacement are <2%^12^) and conservative antibiotic stewardship posits that adjuvant antimicrobials should only be used for challenging established infections,^13^ the open question for HBCS and BCS is if it can eradicate chronic MRSA osteomyelitis. Here, we tested this hypothesis in two rigorous murine models: i) the 1-stage revision septic femoral plate, and ii) the septic transtibial pin.

## Results

### Adjuvant therapy effects of BCA on established implant-associated osteomyelitis following 1-stage revision of a femoral plate in mice

**Fig. 1:**
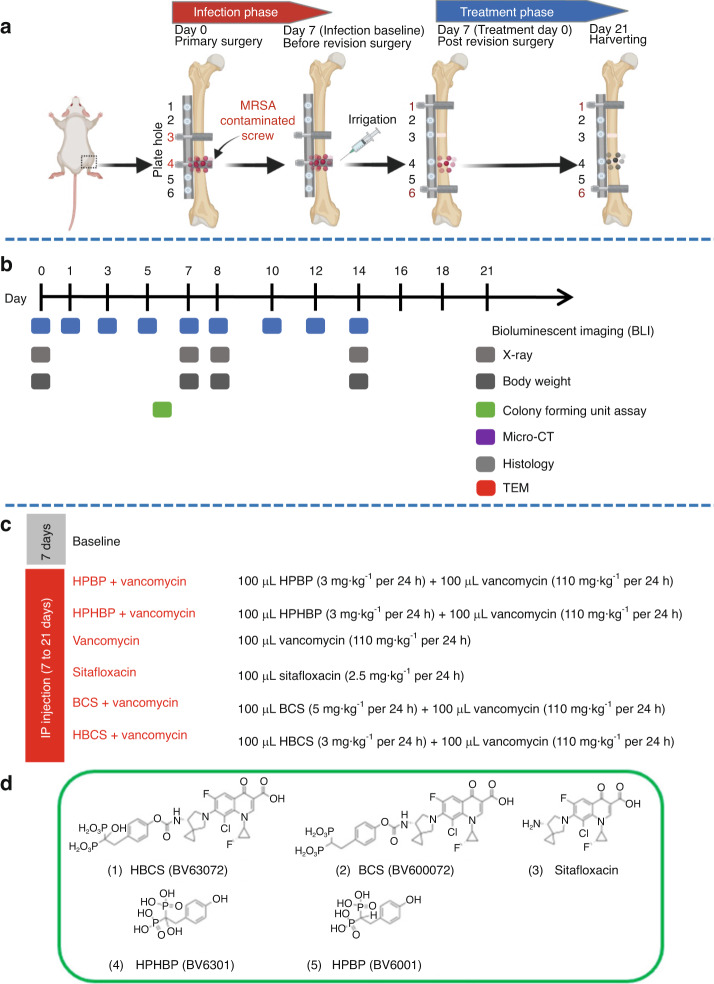
Schematic illustration of murine 1-stage revision model for MRSA implant associated osteomyelitis with outcomes and antibiotic treatments. **a** Osteomyelitis is initiated via surgical implantation of a 6-hole femoral plate that is secured by a sterile screw in hole 3, and a contaminated screw containing ~10^5^ CFU of USA300LAC:Lux in hole 4. On day 7, all implants are removed with debridement and irrigation, a sterile plate is implanted secured by sterile screws in holes 1 and 6 with initiation of antibiotic therapy, and the mice are euthanized on day 21. **b** Timeline of the outcome measures. **c** Seven groups of mice (*n* = 4) were studied. “Baseline” mice were euthanized on day 7 as a control for the infection at the time of revision surgery. The 6 different treatment groups received the indicated i.p. dosing regimen. **d** Molecular structures of the two experimental drugs 1) hydroxybisphosphonate-conjugated sitafloxacin (HBCS, BV63072) and 2) bisphosphonate-conjugated sitafloxacin (BCS, BV600072), 3) parent sitafloxacin to assess effects of non-bone targeted antibiotic, and the bone targeting moieties 4) hydroxyphenylethanehydroxybisphosphonate (HPHBP, BV6301) and 5) hydroxyphenylethanebisphosphonate (HPBP, BV6001) to control for non-nitrogen containing bisphosphonate effects on bone

**Fig. 2:**
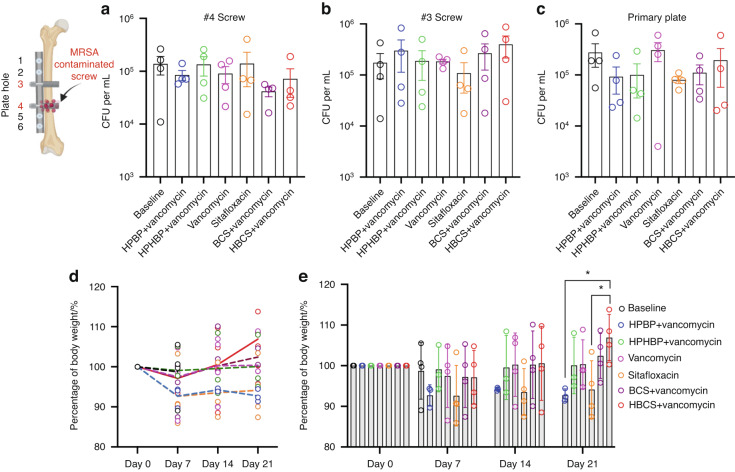
Confirmation of implant-associated osteomyelitis and post-op body weight recovery. **a**–**c** The original screws and femoral plate were removed from the mice on day 7 post-implantation, and the sonicate from each was assessed for bacterial load via CFU assay. Data for each implant are presented with the mean ± SD. No differences between any groups were detected. The body weight for each mouse was determined weekly and reported as the percentage of pre-op body weight for the group (**d** mean over time), and for each mouse with mean ± SD for the group (**e** *P < 0.05 2-way ANOVA)

To assess the adjuvant therapy effects of BCA on top of vancomycin therapy, we utilized an established murine femoral plate 1-stage revision model,^14^ in which mice were infected with bioluminescent MRSA for 7 days to establish chronic osteomyelitis prior to 1-stage revision and randomization into 6 treatment groups based on BLI as described in Fig. 1. Of note is that there were 5 independent control groups: 1) mice euthanized at day 7 to serve as peri-implant osteolysis controls, 2) parenteral vancomycin monotherapy, 3) parenteral sitafloxacin monotherapy, 4) the bone-targeting moiety of BCS, hydroxyphenylethanebisphosphonate (HPBP) plus vancomycin, and 5) the bone-targeting moiety of HBCS, hydroxyphenylethane hydroxybisphosphonate (HPHBP) plus vancomycin. Equivocal implant-associated osteomyelitis was confirmed in all groups via CFU assays of the explanted screws and plates (Fig. 2a–c). We also recorded the weekly body weight for each mouse, which is presented as the % pre-op body weight for each mouse and the group in contiguous line (Fig. 2d) and bar (Fig. 2e) graph format. Interestingly, mice treated with HBCS plus vancomycin was the only group that demonstrated significant increase over their pre-op weight, and had greater weights than mice treated with vancomycin monotherapy at day 21.

**Fig. 3:**
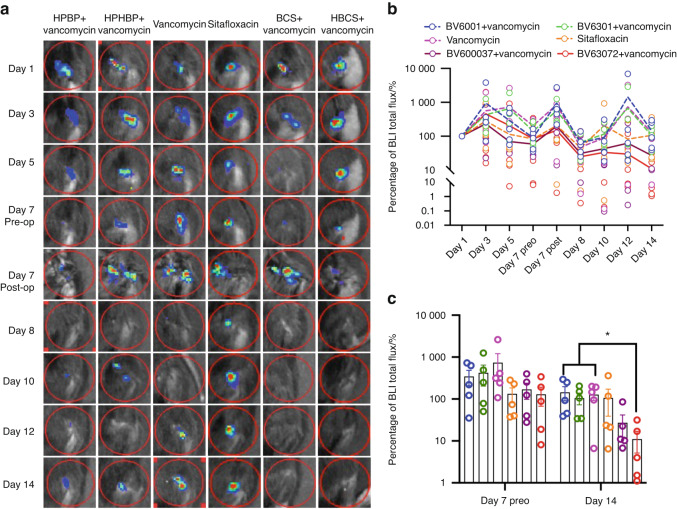
Combination of bisphosphonate-conjugated sitafloxacin and vancomycin therapy eradicate planktonic MRSA following 1-stage revision surgery. Longitudinal in vivo BLI was performed on the indicated day following the original septic implant surgery on day 0, and representative BLI images are shown (**a**), with the longitudinal data presented as the percentage of BLI normalized to day 1 for each group (**b**, mean over time). Unfortunately, several mice were lost to anesthesia and the group sizes for statistical analysis at day 14 are indicated. **c** BLI data for each mouse on day 7 and 14 are presented with mean ± SD for the group (*P < 0.05 Wilcoxon matched-pairs signed rank test)

Longitudinal in vivo BLI was performed to assess BCA therapy on MRSA planktonic growth following revision surgery, and the data are presented in Fig. 3. While the results demonstrated that parenteral sitafloxacin, BCS and HBCS reduced BLI at 12 days, only HBCS treated mice demonstrated a significant decrease in BLI versus vancomycin monotherapy controls 2-weeks post-revision surgery.

**Fig. 4:**
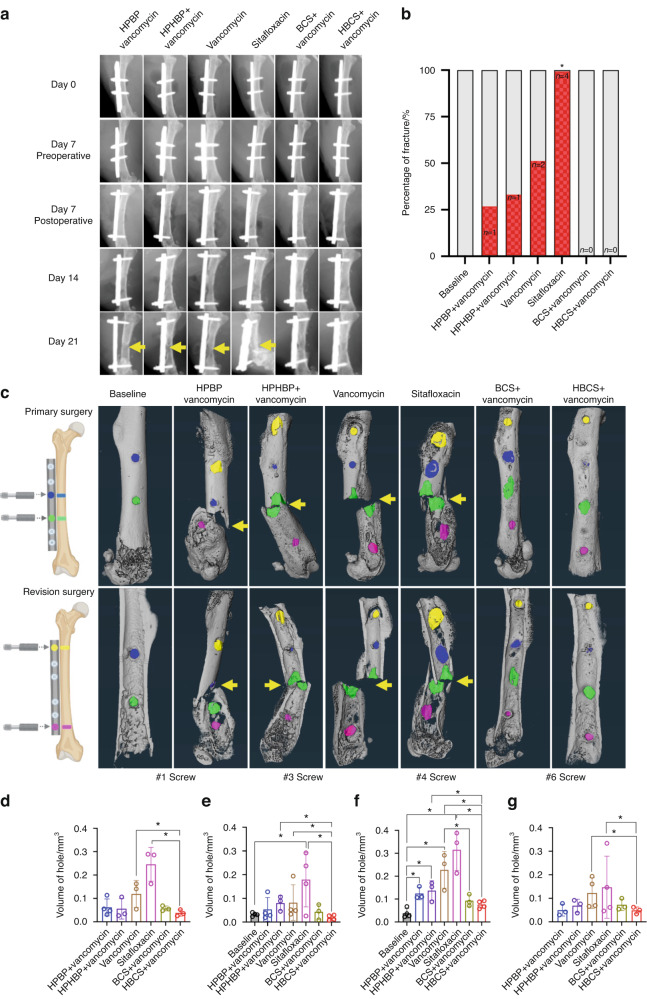
Combination of bisphosphonate-conjugated sitafloxacin and vancomycin therapy prevents catastrophic fractures and peri-implant osteolysis following 1-stage revision surgery. Longitudinal X-rays were obtained on the indicated day following the original septic implant surgery on day 0, and micro-CT was performed on the femurs after harvest on day 21. **a** Representative in vivo X-rays are shown to illustrate the progression of peri-implant osteolysis with Boolean assessment of catastrophic fractures indicated by yellow arrows on day 21 (**b**; *P < 0.05 vs. Baseline via Fishers exact test). **c** The implants were removed for ex vivo volumetric micro-CT of the femurs on day 21, and representative 3D renderings are shown to illustrate the osteolysis area at each screw hole and catastrophic fractures (yellow arrows) observed in the groups that did not receive bisphosphonate-conjugated sitafloxacin. **d**–**g** The volume of each screw hole for each mouse are presented with mean ± SD for the group (*P < 0.05 1-way ANOVA)

As catastrophic femur fracture secondary to peri-implant osteolysis is the most severe adverse event observed in our 1-stage revision model, we assessed this negative outcome via longitudinal X-rays and ex vivo micro-CT (Fig. 4). Assessment of the in vivo day 21 plain films revealed evidence of septic screw hole radiolucency and catastrophic femur fracture originating from this site (#4 screw hole) in all treatment groups except BCS and HBCS treated mice. Remarkably, while none of the vancomycin treated mice demonstrated a significant increase in catastrophic fractures prior to implant removal, all parental sitafloxacin treated mice had radiographic evidence of catastrophic femur fracture on day 21 (Fig. 4b), suggesting that this non-bone-targeted antibiotic has no efficacy against MRSA bone infection. Quantification of the screw hole volumes via ex vivo micro-CT following implant removal confirmed the lack of parenteral sitafloxacin efficacy, and the significant therapeutic effect of HBCS adjuvant therapy on peri-implant osteolysis (Fig. 4c–g).

**Fig. 5:**
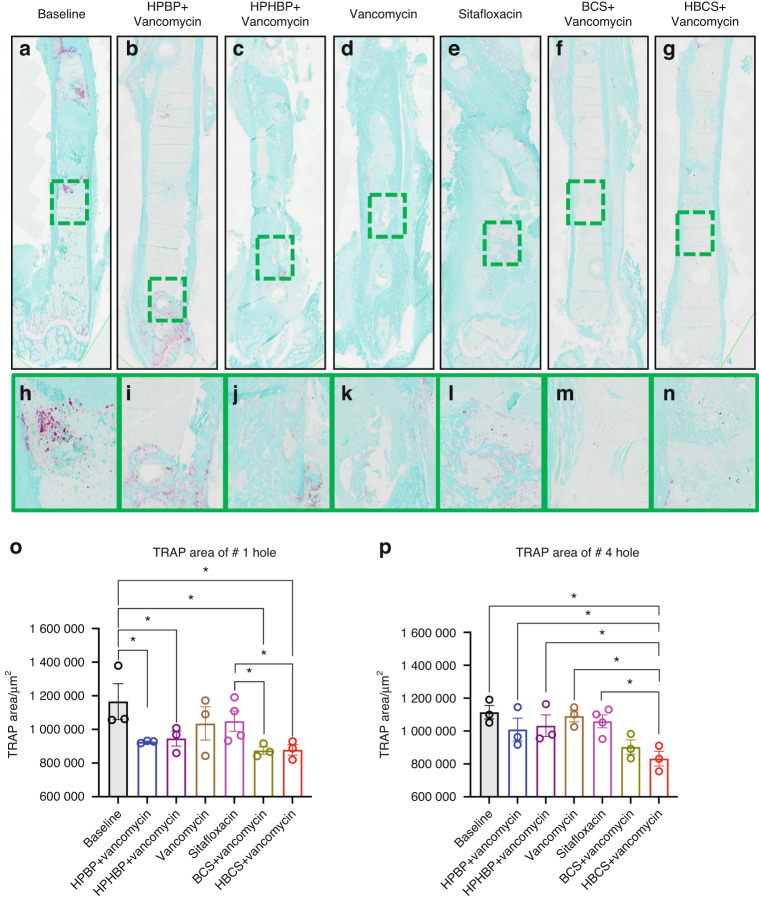
Combination of bisphosphonate-conjugated sitafloxacin and vancomycin therapy reduces peri-implant osteoclast numbers at the site of the screw holes following 1-stage revision surgery. The infected femurs (*n* = 3 or 4) described in Fig. 4 were processed for TRAP-stained histology, and semi-automated histomorphometry was performed to quantify the TRAP^+^ area proximal to the revision screw hole #1, and the original MRSA-infected screw hole #4. Representative x1 images (**a**–**g**) with 40x region of interest (**h**–**n**) are shown to illustrate the number of osteoclasts at the site of bone infection. The TRAP-stained tissue area proximal to screw hole #1 (**o**) and #4 (**p**) was quantified with ImageJ, and the data for each femur is presented with the mean ± SD for the group (*P < 0.05 1-way ANOVA)

**Fig. 6:**
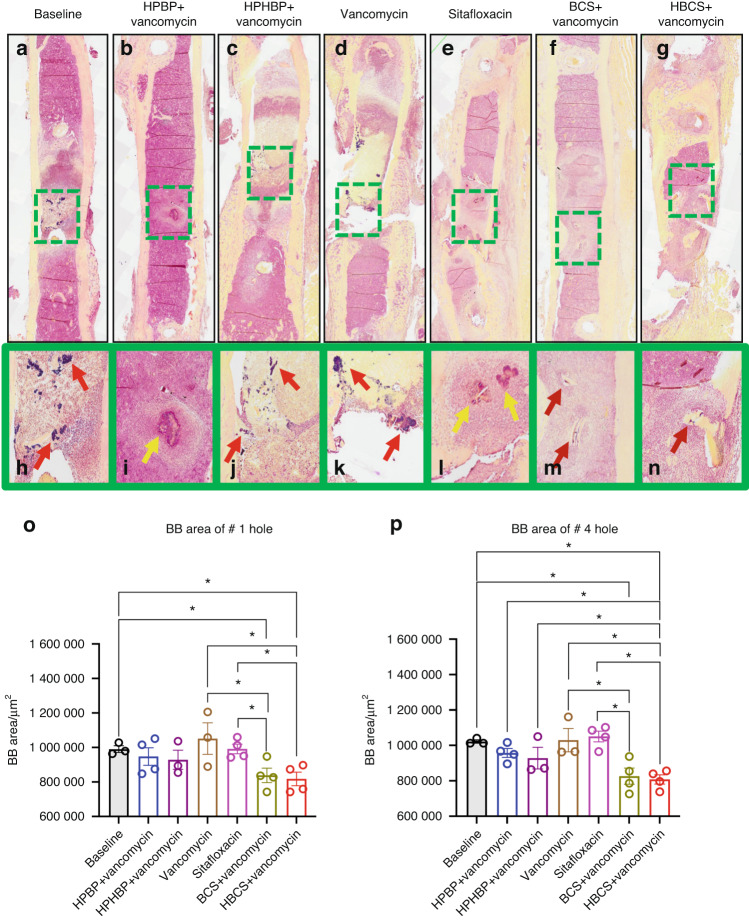
Combination bisphosphonate-conjugated sitafloxacin and vancomycin therapy reduces SACs and Gram^+^ biofilm following 1-stage revision surgery. The infected femurs (*n* = 3 or 4) described in Fig. 4 were processed for Brown and Brenn-stained histology, and semi-automated histomorphometry was performed to quantify the Gram^+^ area proximal to screw holes #1 and #4. Representative 1× images (**a**–**g**) with 5x region of interest (**h**–**n**) are shown to illustrate the SACs (yellow arrows) and bacterial biofilm on bone (red arrows) at the site of bone infection. The Gram-stained tissue area proximal to screw hole #1 (**o**) and #4 (**p**) was quantified with ImageJ, and the data for each femur is presented with the mean ± SD for the group (*P < 0.05 1-way ANOVA)

Although the poor tissue quality of the fractured femurs prohibited rigorous histologic analyses to confirm the antimicrobial and bone protective effects of BCA, we performed a histomorphometry screen for TRAP-stained osteoclasts (Fig. 5) and Gram-positive bacterial biofilm (Fig. 6) on a limited number of samples. The preliminary histology findings demonstrated that adjuvant BCA significantly reduces the area of tissue occupied by osteoclasts and biofilm proximal to the infected screw holes. Taken together with the other results in this study, we concluded that HBCS is the lead candidate BCA based on the trend of superiority over BCS in body weight recovery (Fig. 2e), BLI (Fig. 3c) and peri-implant osteolysis (Fig. 4d–g).

### HBCS monotherapy effects on established MRSA biofilm in a murine transtibial model of implant-associated osteomyelitis

**Fig. 7:**
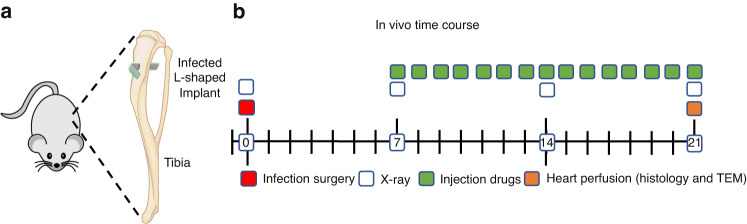
Schematic of transtibial implant model of *S. aureus* implant-associated osteomyelitis with antibiotic therapy. L-shaped wires contaminated with ~10^5^ CFU of USA300LAC:Lux were transcortically implanted through the right tibia (**a**) of 30 mice, which remained untreated for 1 week to established chronic osteomyelitis. On day 7, the mice were randomized into three treatment groups (*n* = 10) : Group 1 Vancomycin (110 mg·kg^−1^ per 24 h); Group 2 Sitafloxacin (2.5 mg·kg^−1^ per 24 h); and Group 3 BV63072 (3 mg·kg^−1^ per 24 h). The infected mice were treated for 14 days, euthanized via perfusion with 4% paraformaldehyde/2.5% glutaraldehyde on day 21 post-op, and the infected tibiae were processed for histology and TEM (**b**)

To overcome the challenges of performing rigorous histologic assessments on fractured femurs and to directly assess the efficacy of our lead BCA candidate vs. SOC antibiotics, we performed a focused study of HBCS vs. parenteral vancomycin and sitafloxacin monotherapy in a well-established septic transtibial pin model in which the mice were infected with a MRSA contaminated pin for 7 days prior to 14 days of treatment as described in Fig. 7. Although this experiment was designed with *n* = 10, some mice were lost to anesthesia and poor tissue quality, which left *n* = 8 in the vancomycin group, *n* = 7 in the sitafloxacin group, and *n* = 8 in the HBCS group (Table 1).

**Table 1:** Histologic outcomes of antibiotic therapy in the murine transtibial implant model of MRSA osteomyelitis

	Vancomycin	Sitafloxacin	HBCS
	Percentage/%	Percentage/%	Percentage/%
Ongoing infection			
Necrotic marrow	8/8 (100%)	5/7 (71.43%)	2/8 (25.00%)**
SACs	8/8 (100%)	4/7 (57.14%)	0/8 (0%)**
Gram positive bone fragements	8/8 (100%)	2/7 (28.57%)*	2/8 (25.00%)**
Endosteal ring of TRAP^+^ osteoclasts	8/8 (100%)	7/7 (100%)	1/8 (0%)**
Infection controlled			
Bridging new bone	0/8 (0%)	2/7 (28.57%)	7/8 (87.50%)**
Ossesous integration	0/8 (0%)	0/8 (0%)	3/8 (37.50%)
Diagnosis (infection controlled or cured)	0/8 (0%)	5/7 (71.43%)**	8/8 (100%)**

**Fig. 8:**
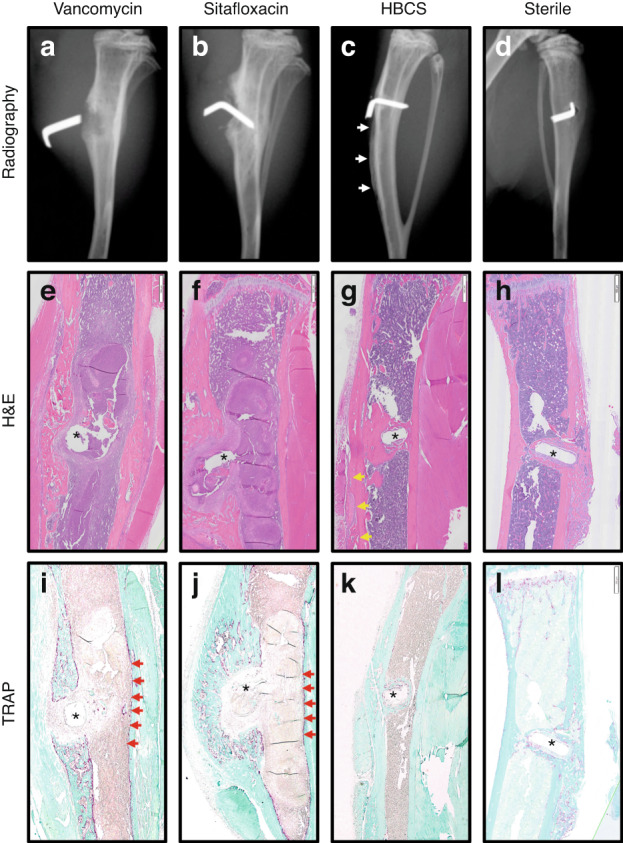
Radiographic and histologic evidence of HBCS-mediated MRSA eradication with implant osseous integration. Representative X-rays, H&E and TRAP-stained histology of the MRSA infected tibiae treated with antibiotics described in Fig. 7 and the sterile pin control are shown to illustrate the radiographic (**a**–**d**) and histologic outcomes of H&E (**e**–**h**), TRAP (**i**–**l**) after 2 weeks of therapy. Note the pin dislocation in the X-rays, and the marrow necrosis with peri-implant osteolysis adjacent to the pinhole (*), observed in 1x images of H&E-stained section from most of the Vancomycin (**e**, **i**) and Sitafloxacin (**f**, **j**) treated tibiae (Table 1), which is consistent with septic implant loosening and chronic osteomyelitis in this model. In contrast, the implant appears to be fixed in the HBCS-treated tibia (**c**, **g**, **k**), there is also healthy bone marrow and new bone formation completely surrounding the flat wire-shaped pinhole (*), which is consistent with the eradication of the infection and osseous integration of the implant, that compared with sterile pin implant (**d**, **h**, **l**)

**Fig. 9:**
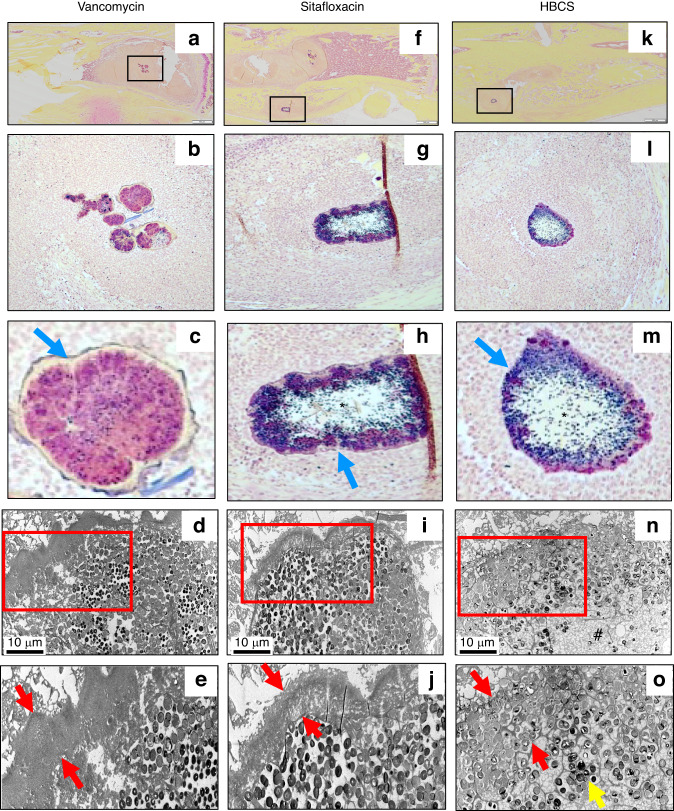
Sitafloxacin kills MRSA within SACs with evidence of fibrin capsule disintegration. To assess antibiotic effects on intramedullary SAC eradication and effects on bacteria morphology, Brown and Brenn-stained histology with subsequent TEM was performed on the infected tibiae described in Fig. 7. Representative images from the Vancomycin (**a**–**e**), Sitafloxacin (**f**–**j**) and HBCS (**k**–**o**) treatment groups are shown. Gram-positive SACs identified at 1x (boxed regions in **a**, **f** and **k**) with corresponding higher power images (**b**, **c**, **g**, **h**, **l** and **m**), and x3 000 TEM (**d**, **i** and **n**) with an enlarged region of interest at x6 000 (**e**, **j** and **o**), are shown. Note the complete lack of vancomycin efficacy, as evidenced by the thick intact fibrin ring (blue arrow in **c** and red arrows in **e**) surrounding the TEM electron-dense bacteria within the SAC. In contrast, sitafloxacin treated SACs contain a necrotic core (*) and a degraded fibrin capsule. These bactericidal effects were more profound in the HBCS treatment Group, as the fibrin capsule was disintegrated, the necrotic core (#) extended all the way to the edge of the SAC, and very few TEM electron-dense bacteria remain (yellow arrow in **o**)

The histology data described in Figs. 8 and 9 were assessed to determine features of infection (necrotic marrow, viable SACs & intramedullary necrotic bone fragments that contained Gram positive biofilm), and absence of infection (new bone bridge the cortices adjacent to the implant, and frank osseous integration of the implant), which were used to derive the diagnosis. Significant differences vs. Vancomycin were assessed via Fisher’s Test (*P < 0.05, **P < 0.01)

Consistent with their lack of efficacy in the femur model, plain radiographs of the infected tibiae prior to sacrifice in mice treated with parenteral vancomycin and sitafloxacin demonstrated extensive peri-implant osteolysis and dislocation of the MRSA contaminated pin (Fig. 8a, b). While similar radiographs were obtained from 5 of the HBCS treated mice, 3 of the animals in this group display remarkable osseointegration of the implant as evidenced by radiodensity around the pin with concomitant reactive cortical bone formation from the initial infection, suggesting that the treatment irradicated the bone infection (Fig. 8c). For comparison purposes, we present an example of aseptic loosening of a transtibial pin from a prior study (Fig. 8d), illustrating the superior outcome achieved in some MRSA infected mice treated with HBCS versus some mice that receive a sterile transtibial pin.

H&E (Fig. 8e–h), TRAP (Fig. 8i–l) and Brown & Brenn (Fig. 9) stained histology confirmed chronic implant-associated osteomyelitis in all vancomycin treated mice as evidenced by the presence of: i) necrotic marrow, ii) viable SACs, iii) intramedullary necrotic bone fragments containing Gram positive biofilm and iv) an endosteal ring of TRAP^+^ osteoclasts; as well as the absence of: v) new bone bridging the cortices adjacent to the implant and vi) frank osseointegration of the implant (Table 1). In contrast, only 5 out of 7 sitafloxacin and all HBCS treated mice had histologic evidence of infection control by these criteria (Table 1). Most remarkable was the frank osseointegration of the implant, as evidenced by new cortical bone completely surrounding the pin hole, whose dimensions are the same as the circumference of the flat wire implant (Fig. 8g).

**Fig. 10:**
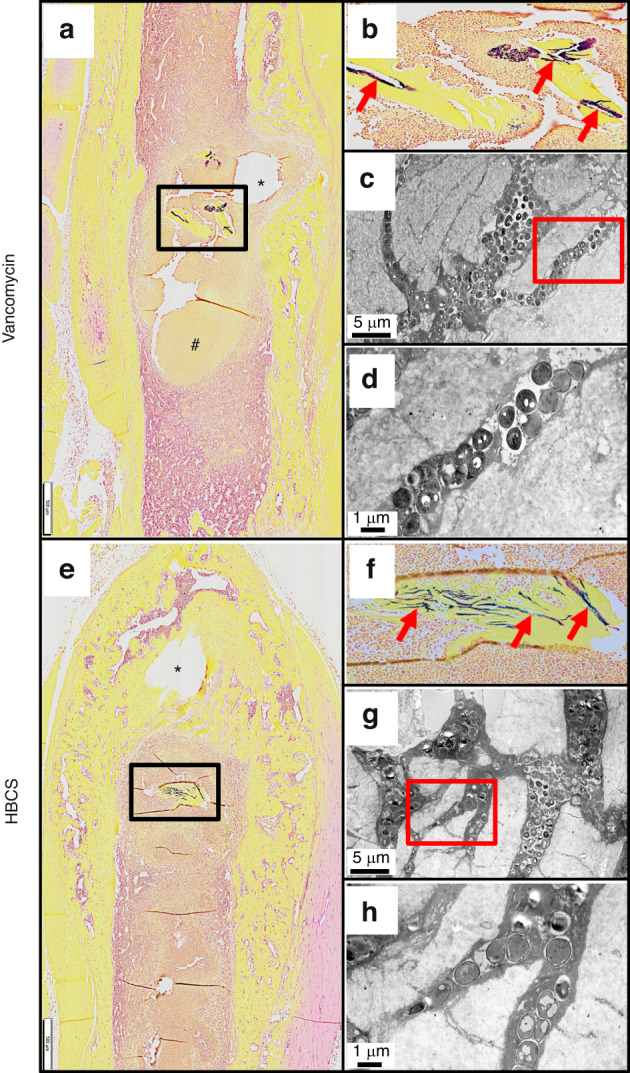
Decreased incidence of MRSA colonization of OLCN in necrotic bone fragments in mice treated with HBCS versus vancomycin treatment control. To assess antibiotic effects on the incidence of MRSA colonization of OLCN and effects on bacteria morphology, histology was analyzed from the mice that received MRSA contaminated transtibial pins described in Fig. 7. Representative 1x images from the Vancomycin (**a**–**d**) and HBCS (**e**–**h**) treatment groups are shown. Gram positive necrotic bone fragments (boxed regions in (**a**, **e)** with corresponding higher power images in (**b**, **f**) highlighting the biofilm (red arrows) were identified in 8 out of 8 tibiae in the Vancomycin treatment group, but only 2 out of 10 tibiae in the HBCS treatment group (Table 1). TEM assessment of this biofilm at x5 000 confirmed MRSA colonization of the OLCN (**c**, **g**), and x15 000 high power images of the boxed regions (**d**, **h**) failed to detect any remarkable differences in bacteria morphology between groups. However, while the marrow necrosis (#) with peri-implant osteolysis as typically seen in this model is observed around the pinhole in the Vancomycin treated tibiae (* in **a**), the pinhole in the HBCS treated tibiae is surrounded by new bone (* in **e**), which may suggest infection control despite the infected necrotic bone fragment in the adjacent marrow space

Brown and Brenn-stained histology and TEM assessment of SACs within the bone marrow confirmed the lack of efficacy for vancomycin, as no effects on the fibrin ring or the bacteria within were noted (Fig. 9a–e). In contrast, both sitafloxacin and HBCS treated tibiae contained SACs with degenerated fibrin rings with necrotic bacteria cores (Fig. 9f–o). In some of the HBCS treated tibiae we observed complete disintegration of the fibrin ring with only a few viable bacteria within (Fig. 9o). These results demonstrate the unique potency of sitafloxacin to eradicate SACs and are consistent with the remarkable MSSA and MRSA killing we previously observed with in vitro biofilms. We also used this Brown and Brenn-stained histology to perform TEM assessment of bacteria within the OLCN of MRSA colonized bone fragments (Fig. 10). The results confirmed the decreased incidence of chronic osteomyelitis detected in HBCS treated mice (Table 1). Interestingly, two mice treated with HBCS contained MRSA colonized bone fragments in their tibiae, and no morphological difference of the bacteria within the OLCN of these samples could be detected versus TEM images of tibiae treated with vancomycin (Fig. 10). Thus, HBCS efficacy against this unique biofilm appears to be an all or none phenomena. However, osseointegration of the septic implant could still be observed in an infected tibia with a colonized necrotic bone fragment (Fig. 10f), which is an unexplained result to be followed up in future investigations.

## Discussion

Implant-associated osteomyelitis remains a major healthcare problem, and current clinical outcomes remain similar to the 77% of 5-year success (survival) rates obtained with SOC implant removal, debridement, and antibiotic therapy first developed in the 1970s.^15^ Moreover, data from the 2018 International Consensus Meeting (ICM) on Musculoskeletal Infections (MSKI) reported no changes in bone infection rates, the primary pathogen, treatment algorithm, and poor outcomes since the SOC treatment algorithm for implant-associated osteomyelitis was established half a century ago.^12,16,17^ However, research in this field has produced several major advances that have refocused our thinking about microbial pathogenesis and the pharmacokinetic and biodistribution requirements of antibiotic therapy for chronic bone infection. Most notable are the discoveries of SACs^18,19^ and *S. aureus* invasion and colonization of the OLCN during chronic osteomyelitis,^20,21^ and that SOC antibiotics (i.e., vancomycin and gentamycin) have little to no effects on these biofilms.^6,7,14,22^ Thus, drug development efforts are needed towards bone-targeted antibiotics that can kill bacteria within mature SACs and OLCN.

Our approach toward overcoming the biodistribution limits of SOC antibiotics for chronic osteomyelitis therapy is to target the drugs directly to the resorbing surface at the bone-bacteria interface via chemical conjugation to bisphosphonate. The motivation for this came from the robust history of safe and effective bisphosphonate therapies, which have proven bone targeting properties with great specificity and minimal side effects on soft tissues.^23^ We have previously demonstrated the utility of this bone-targeting platform with several different types of FDA-approved drugs (e.g., bortezomib^24,25^), and the subject has been extensively reviewed.^23,26–28^ From that success, we aimed to develop a bone-targeted antibiotic conjugate that could achieve sustained concentrations of drug well above the MBEC at the site of bone infection. As an initial proof of concept, we demonstrated that systemic administration of a BP-conjugated fluorophore (AF647-ZOL) specifically labels the cortical surface of the bone in immediate proximity to *S. aureus* bacteria during chronic osteomyelitis,^9^ and that bisphosphonate-conjugated ciprofloxacin has clinical potential for oral indications.^8^ However, since fluoroquinolones are known to have reduced activity against established biofilms, which is particularly true for virulent strains of *S. aureus*,^29,30^ we synthesized a sitafloxacin BCA based on our prior drug library screens for bactericidal activity against *S. aureus* static biofilm and small colony variants.^11,31^ Following our studies demonstrating BCS and HBCS killing of MRSA within biofilms in vitro^9^ and in a prophylaxis model in vivo,^10^ in this study we evaluated their therapeutic efficacy versus SOC antibiotics and non-antibiotic conjugated bisphosphonate controls in a 1-stage revision model.

As longitudinal in vivo BLI of bioluminescent *S. aureus* in mice is known to be a biomarker of planktonic growth during the establishment of implant-associated osteomyelitis^32^ and a tool to demonstrate BCA “target and release” pharmacokinetics in the septic transtibial pin model,^10^ we utilized this approach to assess BCA adjuvant therapy effects on MRSA reactivation following 1-stage revision as previously described in the femoral plate model.^14^ Consistently, we observed post-op spikes in BLI on days 7 and 12 in most of the mice (Fig. 3a, b), confirming MRSA reactivation. While this signal was retained in the control groups, HBCS treated mice displayed a significant decrease in BLI by day 14 versus vancomycin control (Fig. 3c), which was consistent with the significant increase in body weight observed at day 21 (Fig. 2e). Although BCS treated mice displayed a similar trend in efficacy, the results versus vancomycin monotherapy were not significant.

From both a scientific and potential clinical translation standpoint, the most dramatic effect of BCA adjuvant therapy was its complete protection against catastrophic fracture (Fig. 4a, b). For HBCS treated mice, this efficacy was associated with significant decreases in peri-implant osteolysis (Fig. 4c–g), a decrease in osteoclasts proximal to the screw holes (Fig. 5), and a biofilm decrease on necrotic bone fragments proximal to the screw holes versus vancomycin controls (Fig. 6). BCS also demonstrated significant efficacy over vancomycin monotherapy for some of these outcomes, but not as many as HBCS. Thus, although no significant differences were observed between these BCA, we concluded that HBCS was superior to BCS in this 1-stage revision model, likely due to greater binding affinity to bone of the hydroxybisphosphonate component of the HBCS conjugate vs the bisphosphonate component of BCS.^33^

We propose bisphosphonate conjugated-antibiotics (BCA) as an adjuvant therapy following revision surgery of infected orthopedic implants. The septic femoral plate 1-stage revision model is broadly accepted as the best murine model for this purpose. However, as our terminal outcomes are on day 21 post-infection, this proved too challenging for our control groups that did not receive BCA and progressed to catastrophic fractures prior to implant removal (Fig. 4). While this result strongly supports our central hypothesis by demonstrating the superiority of BCA over standard of care vancomycin and free sitafloxacin in a clinically important outcome, the bone tissue was destroyed by the fractures and extensive osteolysis, prohibiting histology studies to confirm our finding and define BCA mechanism of action. Thus, to obtain high quality histology and directly test the effects of HBCS vs. vancomycin and sitafloxacin, we chose to use our transtibial pin model and harvest the infected bone on day 14 post-infection before there was extensive bone loss from fracture and osteomyelitis. This provided excellent histology for histomorphometric analyses as shown in Fig. 8. Although prior studies have demonstrated sitafloxacin’s remarkable efficacy against MRSA biofilm in vitro and in vivo,^9–11^ rigorous head-to-head assessment of its superiority to eradicate established SACs vs. vancomycin has not been reported. Thus, our histologic finding that parental vancomycin has no effects, while parenteral sitafloxacin eradicates SACs via degeneration of the fibrin ring and killing the bacteria within is important (Fig. 9). It is equally remarkable that despite its ability to eradicate SACs, parenteral sitafloxacin had no other efficacy against MRSA as assessed by in vivo BLI, radiology, and histology. However, our finding that the bone-targeting of this drug results in a significantly greater efficacy against reactivation of infection (post-op BLI), greater prevention of catastrophic fractures, and greater eradication of SACs and colonized bone fragments is consistent with our hypothesis that bacteria within the OLCN are not affected by untargeted SOC antibiotics due to biodistribution constraints.

There are several noteworthy limitations to this study, the most apparent being the small number of animals per group in the 1-stage revision model as designed for the screen to identify our lead BCA candidate. Despite these small numbers, we succeeded in demonstrating significant efficacy with our lead conjugate HBCS, warranting expanded studies with HBCS to facilitate its eventual clinical use. Another important limitation is that due to the established infections prior to BCA treatment, these studies could not formally demonstrate that BCS and HBCS reduction of peri-implant osteolysis is secondary to their antimicrobial activity, and not due to direct inhibition of osteoclasts that might be predicted for nitrogen-containing bisphosphonates.^34^ Since BCA comprises a non-nitrogen-containing bisphosphonates known to have minimal effects on bone cells,^9^ it should have no effects on osteoclasts at these concentrations, which is consistent with what we observed in the growth plate of the proximal tibiae adjacent to the infected transtibial pin (Fig. S1). However, since the orientation of these histology sections was optimized for the pin hole, additional studies are needed to further assess the direct effects of HBCS on osteoclasts at sites of bone modeling, remodeling, and repair.

There are also intriguing findings that invite further investigation. Most notably, the osseointegration of MRSA contaminated transtibial implants in HBCS treated mice (Fig. 8c, g, k), suggests that this drug can eradicate implant-associated bone infection with implant retention. More puzzling is our finding of septic implant osseointegration proximal to a MRSA colonized necrotic bone fragment (Fig. 10e–h), suggesting that HBCS treatment allows for a unique sequestrum isolating the infection from adjacent bone formation around the implant. It is also unclear why the HBCS treatment failed to kill the bacteria within this isolated necrotic bone and it defies explanation at this time.

## Conclusions

Here we describe initial evidence of HBCS therapeutic efficacy in murine models of implant-associated osteomyelitis and this drug’s unique ability to prevent catastrophic fractures, eradicate SACs, and mediate osseointegration of septic implants. Follow up studies to confirm these findings in large animals and establish preclinical safety and toxicology are warranted for clinical trial evaluation as a novel drug to treat patients with chronic osteomyelitis.

## Materials and methods

### MRSA strain, in vitro culture, and colony forming units (CFU)

The most prevalent community-acquired MRSA strain is USA300. Thus, we used USA300 LAC::Lux, which has a chromosomal insertion of the bacterial luciferase *lux* gene that allows for in vivo bioluminescent imaging (BLI).^35^ This bioluminescent construct constitutively emits a blue-green light with a maximal emission wavelength of 490 nm only in live and metabolically active bacteria.^36^ The USA300 LAC::Lux bacteria were cultured in tryptic soy broth media at 37 °C overnight as previously described.^9,37^ CFU assays of overnight cultures and sonicated implants were performed as previously described.^14,38^

### Animal surgeries and antibiotics treatments

When we first developed our murine models of implant-associated osteomyelitis we evaluated male and female mice and failed to identify any sexual dimorphisms.^14,32,39^ Thus, since female mice are more cost-effective for research (e.g., 5 mice per cage), easier to handle, and exhibit less aggressive behavior towards post-surgery implant destruction, female mice are the perfect option for our current study. In terms of mouse stains, BALB/c mice are frequently used for a variety of infection studies, mainly because they display TH2-biased immune responses.^40^ All animal experiments were conducted under IACUC-approved protocols. Female BALB/c mice that were 10 weeks old were purchased from Jackson Research Labs (Bar Harbor, ME) and acclimated to a standard microisolator environment for one week prior to surgeries, as previously described.^14,39^ Briefly, the mice were anesthetized with xylazine (20 mg·kg^−1^) and ketamine (100 mg·kg^−1^) administered intraperitoneally, and the right femur or tibia was exposed in accordance with animal surgery guidelines to follow either the *septic femoral plate 1-stage revision model* or *septic transtibial pin model*, respectively, with treatment of bisphosphonate-conjugated sitafloxacin (BCS) and hydroxybisphosphonate-conjugate sitafloxacin (HBCS) as previous descripted.^9,10^

#### Experiment 1: The septic femoral plate 1-stage revision model

A total of 28 mice underwent septic femoral plate surgery as described in Fig. 1. The right femur was implanted with a 6-hole femoral titanium plate and screws (RISystem, Davos, Switzerland) that was secured by a sterile screw in hole ^#^3 and a screw containing ~10^5^ CFU of USA300LAC::Lux in hole ^#^4. The presence of osteomyelitis was confirmed via X-ray, BLI, and CFU assays on day 7 as previously described.^14^ Also on day 7, all implants were removed with debridement and irrigation, and a sterile plate was implanted and secured by sterile screws in holes ^#^1 and ^#^6 with the initiation of antibiotic therapy (Fig. 1a, b). To verify the efficacy of SOC with bisphosphonate-conjugated sitafloxacin (BCS) and hydroxybisphosphonate-conjugated sitafloxacin (HBCS), the mice were randomized into seven treatment groups: 1) Baseline (day 7 post-op to serve as a cross-section control for peri-implant osteolysis) (*n* = 4); 2) Hydroxyphenylethanebisphosphonate (HPBP, BV6001) + Vancomycin (*n* = 4); 3) Hydroxyphenylethanehydroxybisphosphonate (HPHBP, BV6301) + Vancomycin (*n* = 4); 4) Vancomycin (*n* = 4); 5) Sitafloxacin(*n* = 4); 6) BCS (BV600072) + Vancomycin (*n* = 4); 7) HBCS (BV63072) + Vancomycin (*n* = 4). The parent sitafloxacin was used to assess the effects of non-bone targeted antibiotics, while HPHBP and HPBP were used as control for non-nitrogen-containing bisphosphonate effects on bone, and the mice were euthanized on day 21(Fig. 1c). The chemical structures and commercial product numbers of these compounds are shown in Fig. 1d.

#### Experiment 2: Septic transtibial pin model

To further assess the antimicrobial efficacy of HBCS and its mechanism of action, a transtibial pin model was employed as previously described.^39^ Briefly, the right tibia was shaved, and the skin was cleansed with 70% ethanol. Infection was initiated by placing a stainless steel L-shape pin contaminated with ~10^5^ CFU of USA300LAC::Lux transcortical through the tibia via medical to lateral implantation which remained untreated for 1 week to establish chronic osteomyelitis (Fig. 7a). On day 7, the mice were randomized into three monotherapy treatment groups (*n* = 10 per group): 1) Vancomycin (110 mg·kg^−1^ per 24 h); 2) Sitafloxacin (2.5 mg·kg^−1^ per 24 h); and 3) HBCS (BV63072) (3 mg·kg^−1^ per 24 h). The infected mice were treated for another 14 days, euthanized via heart perfusion with 4% paraformaldehyde/2.5% glutaraldehyde on day 21, and the infected tibiae were processed for histology and TEM (Fig. 7b).

### Bioluminescence imaging

For mice in Experiment 1, in vivo BLI was performed on days 0, 1, 3, 5, 7, 7 (postoperative), 8, 10, 12 and 14 as indicated in Fig. 1. Prior to performing the BLI, the mice were anesthetized with ketamine/xylazine (100 mg/20 mg/kg) and the femur with implant was included in the region of interest (ROI) for imaging and analysis. The BLI data are presented on a color-scale overlapped with a grayscale photograph of the femur and quantified as total flux (photons/s) within a standardized circular ROI using Living Image software (Caliper).^41^ Of note is that absence of BLI signal on day 1 is an exclusion criteria for this outcome measure, as the data at later timepoints are normalized to this value.^14^ Thus, as seven mice had no BLI signal on day 1 post-op, BLI analyses for all treatment groups were reduced to *n* = 3. Following randomization into treatment groups, one mouse in Group 2 and one mouse in Group 3 died due to accidental overdose of anesthesia. In order to meet the requirements for statistical analysis, we did another set of BLI experiments to make the sample size of each group reach *n* = 5 (Fig. 3c).

### Radiographic and Micro-CT

Infected femurs were harvested on day 21 in experiment 1, peri-implant osteolysis and osseointegration was assessed radiographically using a Faxitron Cabinet X-ray system (Faxitron, Wheeling, IL, USA) as previously described.^42^ Micro-CT was performed to quantify screw hole volume as previously described.^41,43^

### Histology

Infected long bones were dissected to remove the implants, decalcified, and embedded in paraffin. At the mid-point of the femur or tibia on the sagittal cuts, five 5 µm tissue sections were obtained from three levels and mounted onto slides. One slide per orientation of cut was stained with H&E, Brown-Brenn, or for tartrate-resistant acid phosphatase (TRAP) as previously described.^10^ Semi-automated histomorphometry to quantify biofilm area and TRAP^+^ area was performed with Visiopharm software as previously described.^10^

### Transmission electron microscopy (TEM)

For mice in Experiment 2, regions of interest (ROI) within serial sectioned paraffin blocks of infected tibia samples were identified from the Brown-Brenn stained sections, and adjacent unstained slides were reprocessed for TEM using the “pop-off” technique, as previously described.^44^ Slides were deparaffinized in 3 changes xylene and then rehydrated through a graded series of ethanol back to ddH_2_O. Rehydrated sections on slides were post fixed overnight at 4 °C in 0.1 mol·L^−^^1^ phosphate buffered 2.5% glutaraldehyde, then 1% OsO_4_ for 20 min at room temperature. The slides were washed in ddH_2_O, dehydrated in a graded series ethanol to 100% (x3), infiltrated with a 1:1 mixture of 100% ethanol and Spurr’s resin, then 100% Spurr’s resin overnight at room temperature. The ROI (etched with a diamond pen on the back side of the slides) was re-identified after the slides were drained of excess resin. A size 3 BEEM capsule filled with fresh resin was placed over the paraffin section’s ROI and polymerized 24 h at 65˚C. BEEM capsules, with entrapped ROIs, were popped off slides by dipping them 5–10 s rapidly in liquid nitrogen. The popped off block was trimmed to the ROI, thin sectioned at ~70 nm, and placed onto formvar carbon coated nickel slot grids for imaging using a Gatan Erlangshen 11-megapixel digital camera (Pleasanton, CA) and a Hitachi 7650 TEM (Pleasanton, CA).

### Statistics

CFU data were quantified for each implant to determine the mean ± SD for the group, and statistical significance was assessed by 1-way ANOVA. Percent of body weight from pre-op day 0 was quantified for each mouse to determine the mean ± SD for the group, and statistical significance was assessed by 2-way ANOVA. Longitudinal BLI data are presented as the % BLI normalized to day 1 for each group and for each mouse with mean ± SD for the group, and statistical significance was assessed by Wilcoxon matched-pairs signed rank test. Boolean assessment of catastrophic fractures was determined from X-ray prior to removal of the implants by Fisher’s exact test. Micro-CT assessment of screw hole volume was determined for each mouse with mean ± SD for the group, and statistical significance was assessed by 1-way ANOVA. Histomorphometric quantification of biofilm and TRAP^+^ area was determined with ImageJ for each mouse with mean ± SD for the group, and statistical significance was assessed by 1-way ANOVA. Boolean assessments of ongoing infection (presence of necrotic marrow, viable SACs, Gram positive bone fragments, and endosteal ring of osteoclasts) and infection control (presence of bridging new bone and osseous integration of the implant) were quantified as the percentage of animals per group and significant differences from vancomycin control were assessed by Fisher’s exact test.

### Supplementary information

Supplementary Figure 1
