# Supplementary figures and images for: Evidence of bisphosphonate-conjugated sitafloxacin eradication of established methicillin-resistant S. aureus infection with osseointegration in murine models of implant-associated osteomyelitis
Source: Bone Res. 2023 Oct 18;11:51. doi: 10.1038/s41413-023-00287-4 (PMC10582111; doi:10.1038/s41413-023-00287-4)

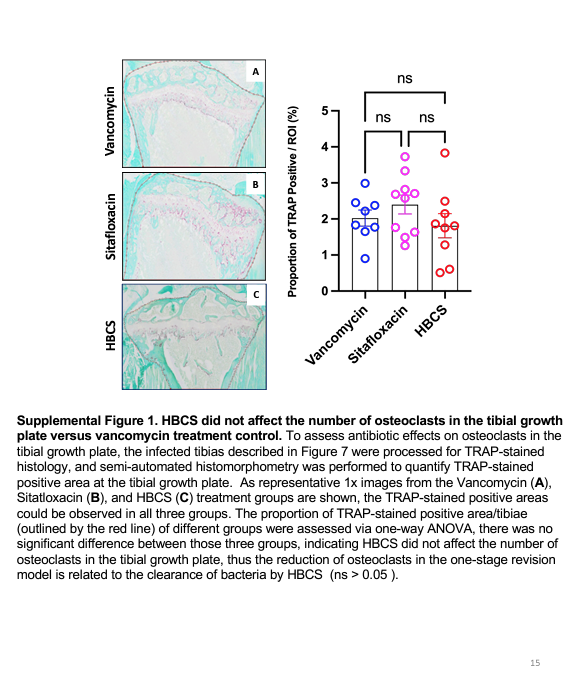

Supplement: Supplementary file 1 — Supplementary Figure 1 [file 41413_2023_287_MOESM1_ESM.png]
